# The assessment of decision-making ability in the forensic evaluation of capacity for civil conduct in patients with schizophrenia

**DOI:** 10.1192/j.eurpsy.2025.1518

**Published:** 2025-08-26

**Authors:** W. Li

**Affiliations:** 1 Department of Forensic Psychiatry, Academy of Forensic Sciences, Shanghai, China

## Abstract

**Introduction:**

Patients with schizophrenia often require civil capacity assessments when participating in civil activities. The primary focus of the assessment involves evaluating patients’ understanding of the issues at hand, their awareness of potential choices and the corresponding outcomes, and their ability to make decisions after considering the advantages and disadvantages. As such, the ability to make decisions is the fundamental neuropsychological mechanism underlying civil activities.

**Objectives:**

This study systematically reviewed existing research on decision-making ability in patients with schizophrenia.

**Methods:**

Both major international and Chinese databases were systematically searched. Relevant studies were summarized in aspects of the assessment, neuropsychological mechanisms, and neurobiological mechanisms of decision-making ability in patients with schizophrenia.

**Results:**

1. The most frequently employed experimental paradigms in studies focusing on economic decision-making include the Iowa Gambling Task (IGT) and the Game of Dice Task (GDT). Patients with schizophrenia performed significantly worse on the IGT compared to healthy individuals, often overestimating immediate gains and losses while failing to learn from the frequency of wins. There are relatively few studies utilizing the GDT, and the findings are inconsistent across studies.
2. Cognitive domains related to the decision-making ability in patients with schizophrenia could be executive function, verbal memory, and working memory. Psychiatric symptoms related to the decision-making ability include diminished motivation, lack of interest, depressive symptoms, and negative symptoms. Moreover, emotion plays a critical role in decision-making behaviors. Decision-making ability can also be influenced by medication and the overall severity of the illness; however, some studies found no association between decision-making ability and psychiatric symptoms, the illness stage, or medication usage.
3. Imaging studies consistently indicate that the prefrontal cortex is a critical brain region associated with decision-making abilities. Brain areas such as the orbitofrontal and ventromedial prefrontal cortex, amygdala, frontoparietal cortex, medial prefrontal cortex, dorsomedial prefrontal cortex, bilateral thalamus, and the left dorsal anterior cingulate cortex may play a role in decision-making processes in patients with schizophrenia. Nonetheless, some research found no association between decision-making ability and the functioning of the dorsomedial prefrontal cortex.

**Conclusions:**

Deeply exploring the neuropsychological and neurophysiological mechanisms behind decision-making ability can help the understanding of the decision-making behavior of patients with schizophrenia in civil activities and can benefit forensic evaluation of civil capacity.

**Disclosure of Interest:**

None Declared

